# Supplementary material for: Establishment of titration-based control of DNA replication in Escherichia coli
Source: Sci Adv. 2026 Oct 16;12(42):eaei9788. doi: 10.1126/sciadv.aei9788 (PMC13674477; doi:10.1126/sciadv.aei9788)
Supplement: Supplementary file 1 [file sciadv.aei9788_sm.pdf]

Science Advances
AAAS

Supplementary Materials for

Establishment of titration-based control of DNA replication in *Escherichia coli*

Belén Adiego-Pérez *et al.*

Corresponding author: Lorenzo Olivi, lorenzo.olivi@icm.uu.se

Sci. Adv. **12**, eaei9788 (2026)
DOI: 10.1126/sciadv.aei9788

The PDF file includes:

Figs. S1 to S9
Tables S1 to S7
Legends for data S1

Other Supplementary Material for this manuscript includes the following:

Data S1

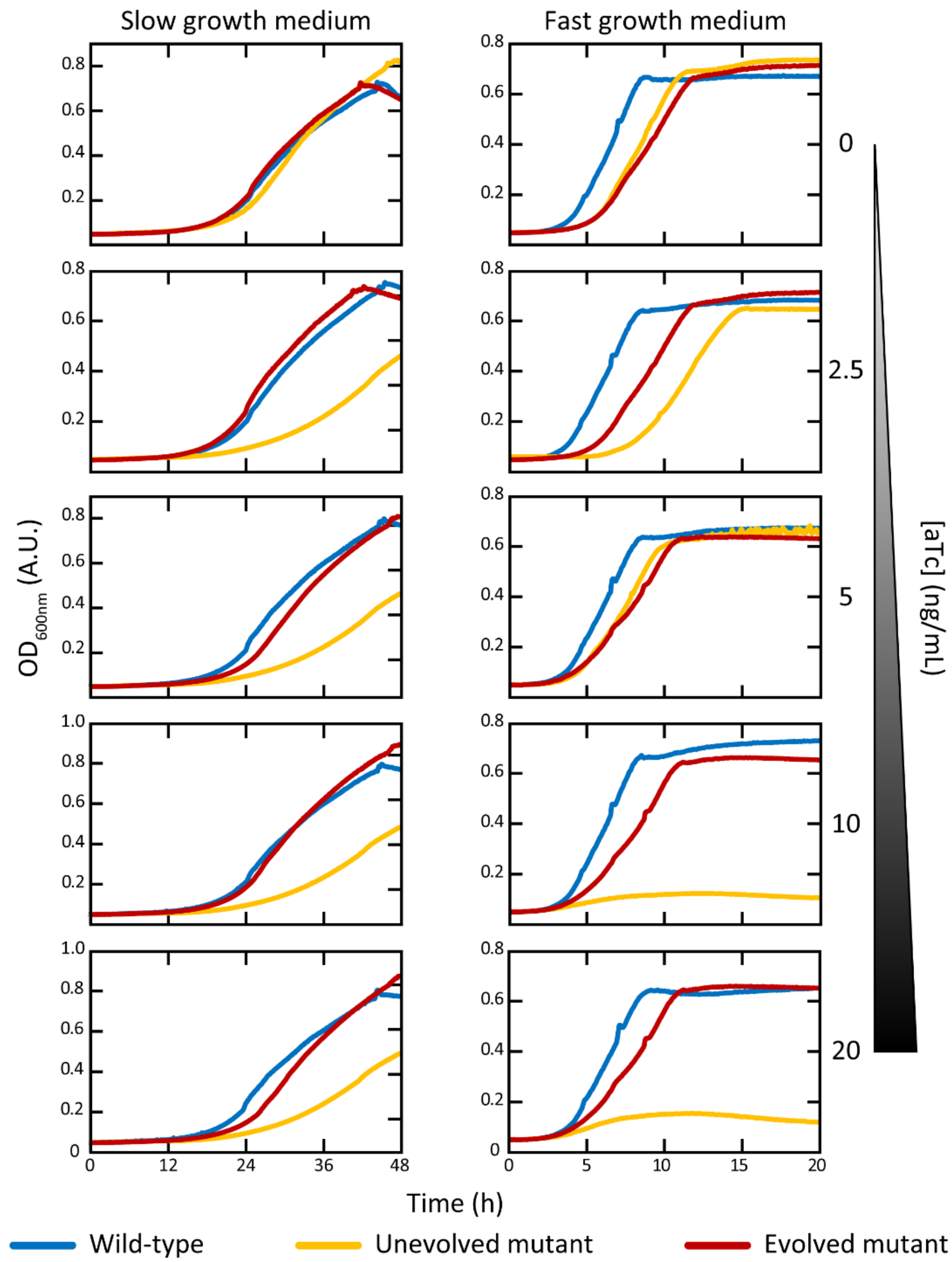

Figure S1. Growth phenotype of *E. coli* mutant strains. Growth characterisation of the wild-type *E. coli* MG1655 (blue line), the original mutant strain carrying the $p_{LtetO-1}$-*tetR-dnaAcos* locus (yellow line) and the mutant strain after evolution in fast growth and 10 ng/mL of aTc (red line). Cells were grown in either slow growth (left) or fast growth medium (right), each supplemented with five concentrations of aTc (0, 2.5, 5, 10 and 20 ng/mL).

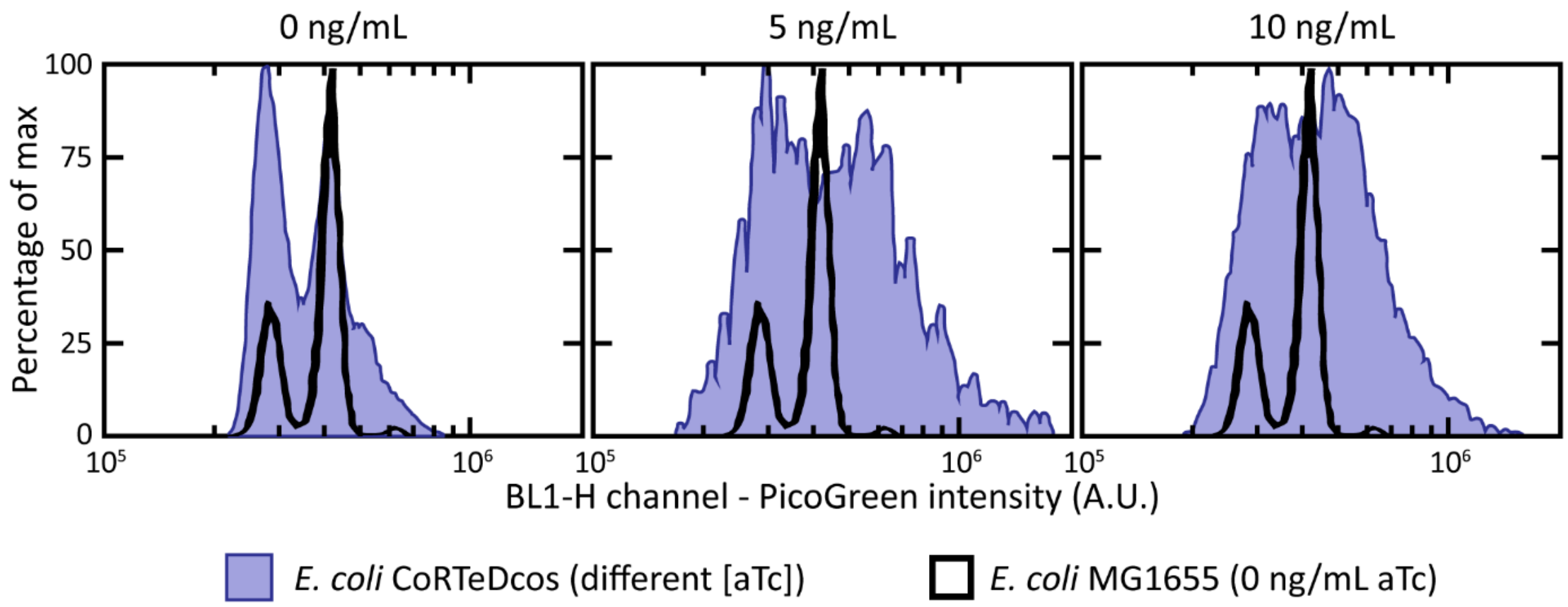

Figure S2. *E. coli* CoRTeDcos is resistant to rifampicin treatment. To obtain a measure of the average number of origins of replication in a *E. coli* population, cells were treated with rifampicin and cephalexin for six hours. This treatment normally stops new initiation events, allows any ongoing replication round to finish and prevents cell division. As a result, population of wild-type *E. coli* cells normally arrange in discrete peaks, with each peak indicating an integer increment in number of origins (black lines). On the contrary, slow-growing ($\lambda \approx 0.3$ h^{-1}) *E. coli* CoRTeDcos cells showed a less defined division of populations, a behaviour that worsened at progressively higher levels of induction (left: 0 ng/mL aTc; middle 5 ng/mL aTc; right: 10 ng/mL aTc). Whereas there was an increase in DNA content between conditions, the histograms did not represent synchronised populations where cells stopped replicating in the time frame of antibiotic treatment.

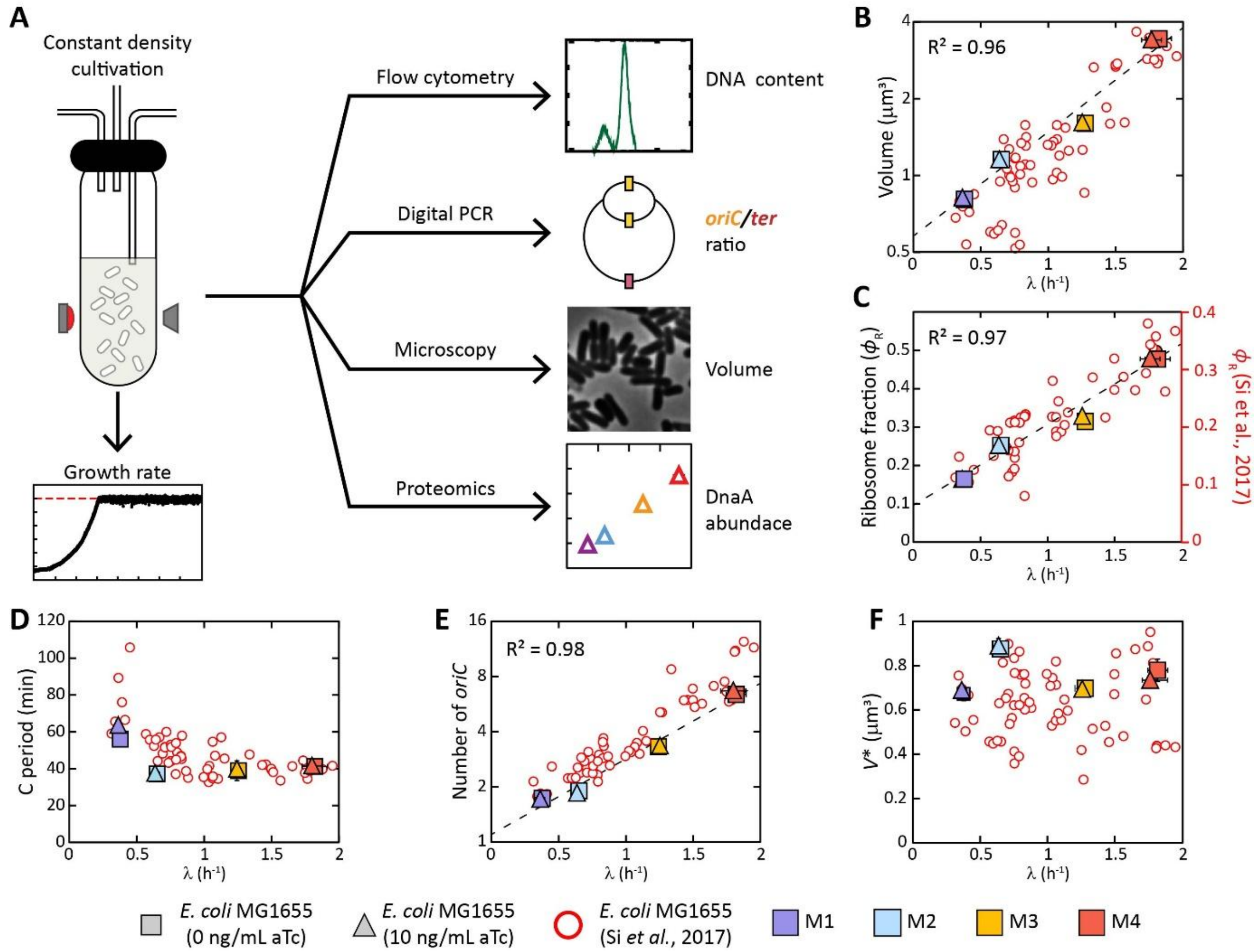

Figure S3. Validation of our cultivation approach. A) In our set-up, cell growth is balanced in exponential phase inside turbidostat reactors, maintaining the turbidity of the cell culture at a constant OD_{600nm} = 0.3. After 10 generations, cells are harvested in different aliquots to be subjected to either flow cytometry for DNA content measurements, digital PCR for determination of *oriC/ter* ratio, microscopy for volume measurements or quantitative proteomics. We validated our cultivation strategy by recreating quantitative behaviours of *E. coli*, such as **B)** increase of volume with growth rate, **C)** increase of proteome ribosome fraction over growth rate, **D)** stable C period times, **E)** increase of the number of origins of replication over growth rate and **F)** stable volumes of initiation over growth rate. Data points are average of replicates and standard deviations. Data from (7) are plotted for validation.

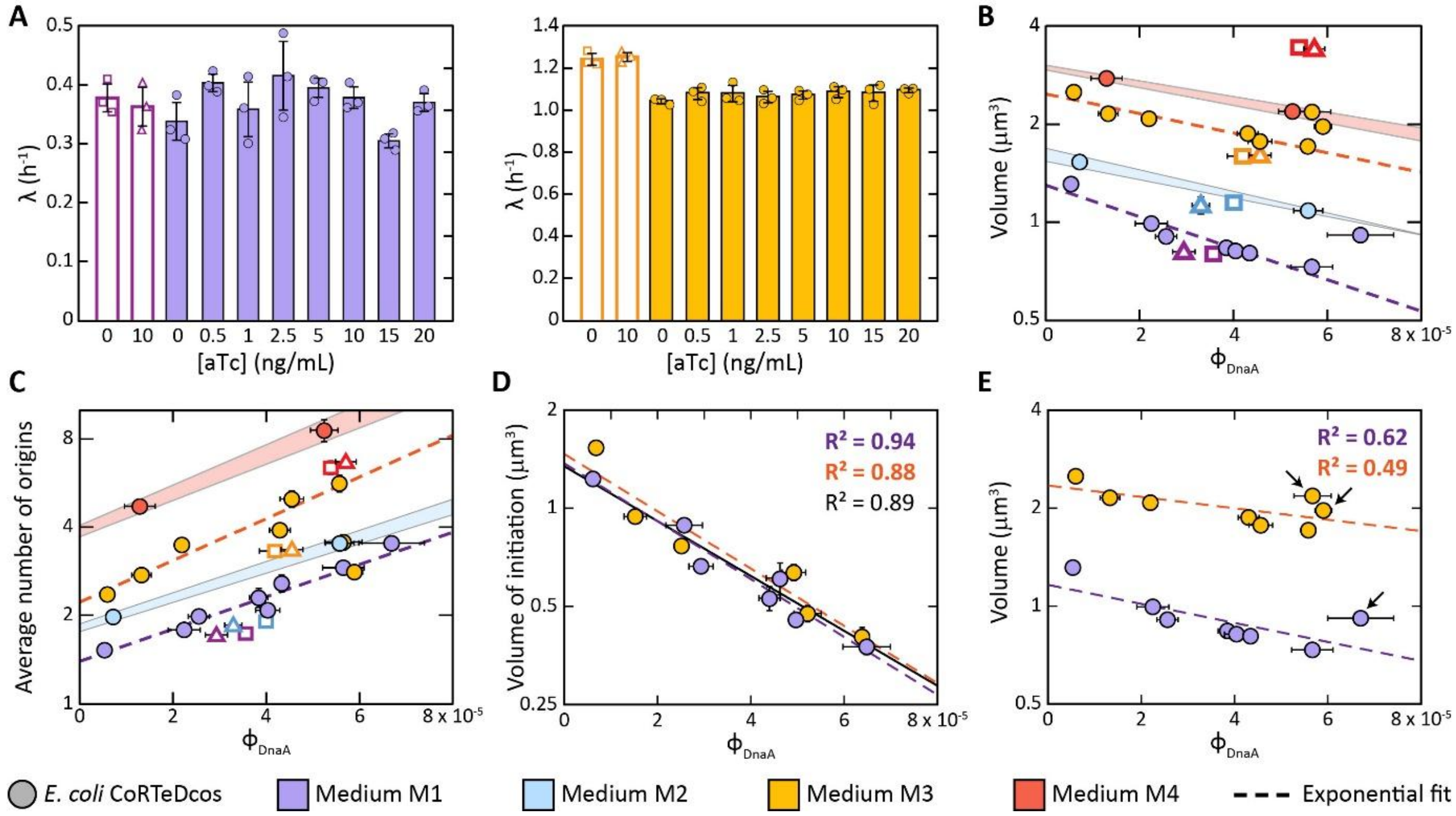

Figure S4. Inducer-dependent behaviour of *E. coli* CoRTeDcos. A) Different levels of inducer do not impact growth rate in *E. coli* CoRTeDcos in medium M1 and M3. See Figure 2 in the main text for the effect of aTc on the growth of *E. coli* CoRTeDcos in medium M2 and M4. **B)** Volume and **C)** number of origins of replication of *E. coli* CoRTeDcos at different DnaA mass fraction (ϕ_{DnaA}) in all media. For media M1 and M3, the exponential relationships are the same as in the main text (Figure 3B). For media M2 and M4, the shaded regions represent hypothetical trends, based on the available measurements and their standard deviations. **D)** Individual exponential relationships between ϕ_{DnaA} and volume of initiation in media M1 and M3. The exponential relationship derived from *E. coli* CoRTeDcos in all media and inducer concentrations (black continuous line) is the same as in the main text (Figure 3D). **E)** Trendlines of the relationship between ϕ_{DnaA} and volume for *E. coli* CoRTeDcos in media M1 and M3 including measurements coming from all inducer concentrations. Measurements from cell grown in medium M1 with 20 ng/mL aTc and in medium M3 with either 15 or 20 ng/mL aTc (indicated by arrows) deviate from the general exponential relationship. They were considered as outliers and excluded from the exponential fits of the main text. Purple, blue, yellow, and red symbols represent data acquired from cells grown in media M1, M2, M3 and M4, respectively. All data points are the average of three independent biological replicates and error bars represent the standard deviation. In some cases, error bars are smaller than the symbols. In **A)**, individual data points are plotted.

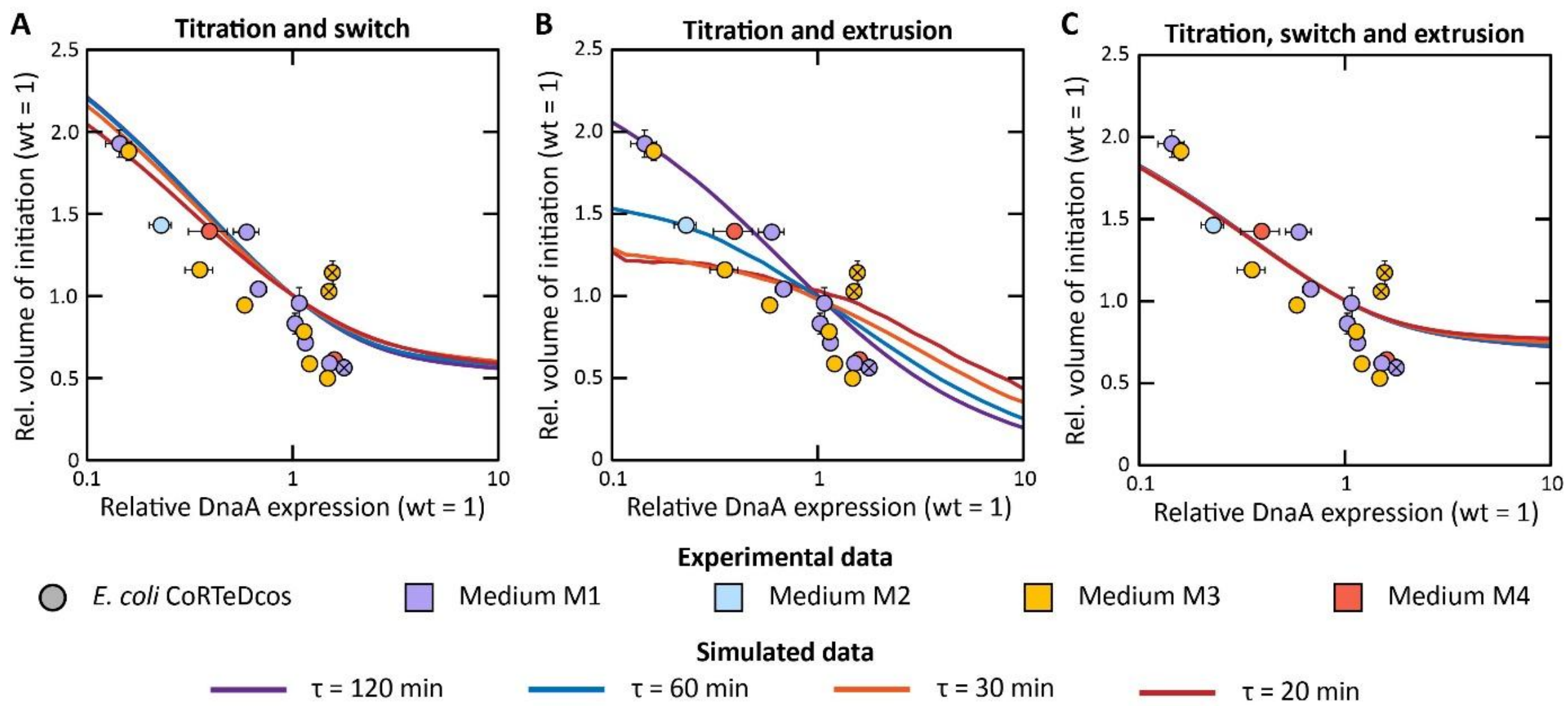

Figure S5. Comparison of experimental *E. coli* CoRTeDcos data with different models of *E. coli* DNA replication initiation. The experimental relationship between the relative expression of DnaA and the relative volume of initiation (normalised for the average of all values in the same medium) is compared with prediction coming from simulations of *E. coli* DNA replication using different models: **A)** titration of DnaA and DnaA-ATP/DnaA-ADP interconversion ($\chi_\nu^2 \approx 45$); **B)** titration of DnaA and extrusion modulated by H-NS ($\chi_\nu^2 \approx 71$); **C)** titration of DnaA, DnaA-ATP/DnaA-ADP interconversion and extrusion modulated by H-NS ($\chi_\nu^2 \approx 54$). Purple, blue, yellow, and red symbols indicate experimental data collected in medium M1, M2, M3 and M4, respectively. Purple, blue, orange, and red solid lines indicate simulated trends for cells with doubling times of 120 min, 60 min, 30 min and 20 min, respectively. All data points are the average of three independent biological replicates and error bars represent the standard deviation. In some cases, error bars are smaller than the symbols.

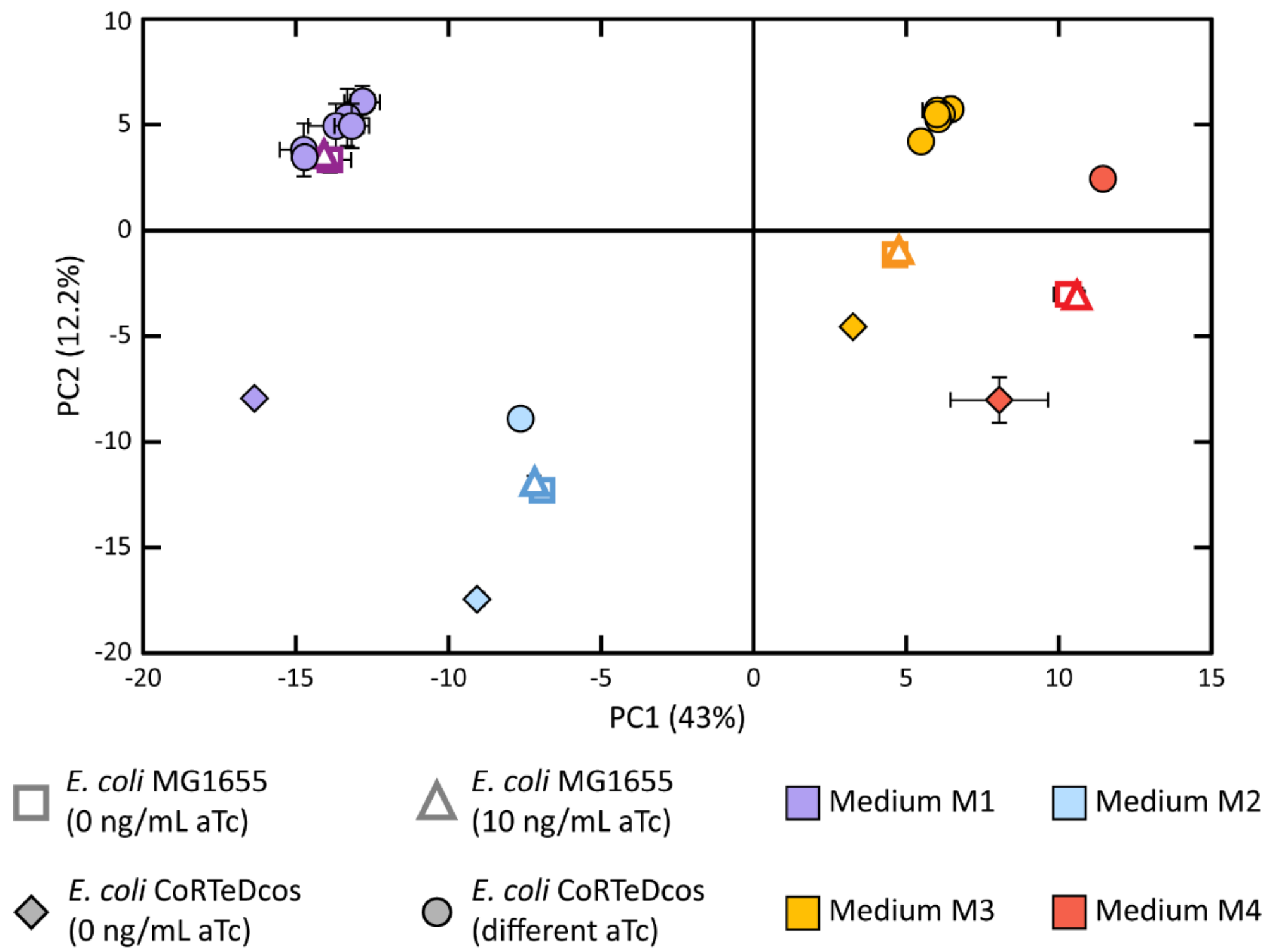

Figure S6. Principal component analysis of the proteomes of *E. coli* MG1655 and *E. coli* CoRTeDcos in all the tested conditions. In all growth conditions, the proteome of *E. coli* CoRTeDcos only minorly changed in the presence of different concentrations of aTc (filled circles), but it was majorly altered in the absence of the inducer (filled diamonds). On the other hand, the proteome of *E. coli* MG1655 remained unchanged in either absence (empty squares) or presence of aTc (empty triangles), indicating that all the changes observed in *E. coli* CoRTeDcos come from the different expression levels of DnaAcos.

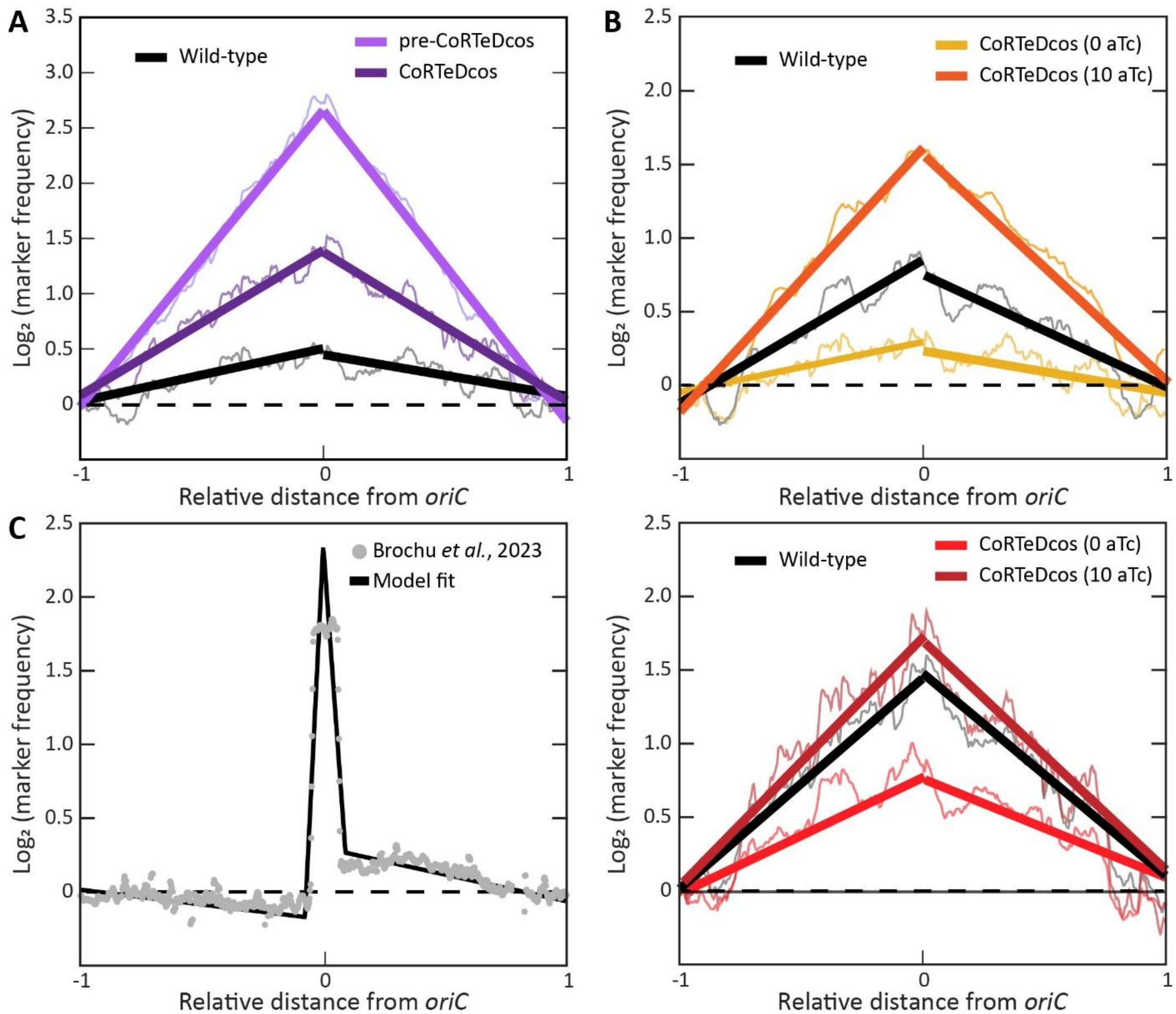

Figure S7. Marker frequency analysis of *E. coli* MG1655, *E. coli* pre-CoRTeDcos and *E. coli* CoRTeDcos. A) In medium M1 ($\lambda \approx 0.3$ h^{-1}) and in presence of 10 ng/mL of aTc, *E. coli* pre-CoRTeDcos (light purple) shows a higher origin/terminus ratio than *E. coli* CoRTeDcos (dark purple), hinting at an effect of DnaB(M242I) in reducing initiation of DNA replication. Both strains have a higher origin/terminus than *E. coli* MG1655. **B)** Marker frequency analysis of *E. coli* CoRTeDcos grown in media M3 (top) and M4 (bottom) either with 0 ng/mL or 10 ng/mL of aTc, compared to *E. coli* MG1655. The decay of marker frequency of each replichore is fitted to either a straight line, indicative of normal fork progression, or a broken-stick regression, indicative of replication fork arrest, and the best fit is then displayed (bold line). In all the tested conditions, we did not observe any geometric feature indicative of fork attrition or arrest. **C)** The approach used to infer presence of replication fork attrition is benchmarked against sequencing reads coming from an *E. coli* strain with a known non-linear marker frequency profile (96). In all panels, marker frequencies are normalized to the terminus of replication. In panel A and B, thin lines indicate the trendlines of marker frequency (moving average of 60 bins), thick lines indicate the best-fitting model, and black lines indicate *E. coli* MG1655. In panel C, dots correspond to the total read count of a 5000 bp fragment of the genome.

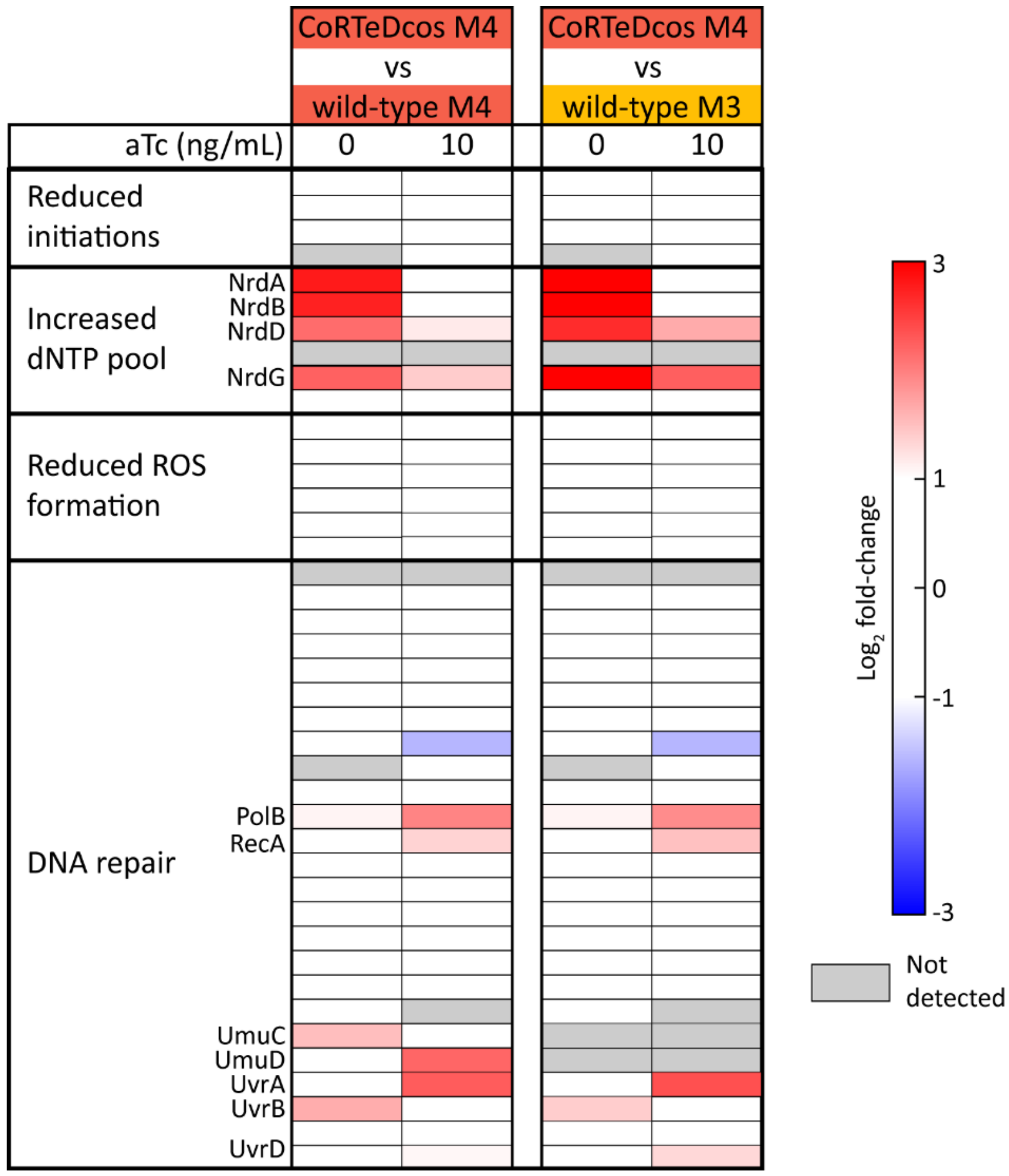

Figure S8. Replicative stress in *E. coli* CoRTeDcos during fast growth. The proteome of *E. coli* CoRTeDcos grown in medium M4 either with 0 ng/mL aTc ($\lambda \approx 1.46\ h^{-1}$) or 10 ng/mL aTc ($\lambda \approx 1.28\ h^{-1}$) is compared to the one of *E. coli* MG1655 grown in medium M4 ($\lambda \approx 1.83\ h^{-1}$) (same as main text Fig. 5) or in medium M3 ($\lambda \approx 1.25\ h^{-1}$) to exclude growth rate-dependent changes in expression. The same proteins are found to be differently expressed independently of the tested medium. The complete list of proteins belonging to each group is available in Data S1, together with their relative fold-change.

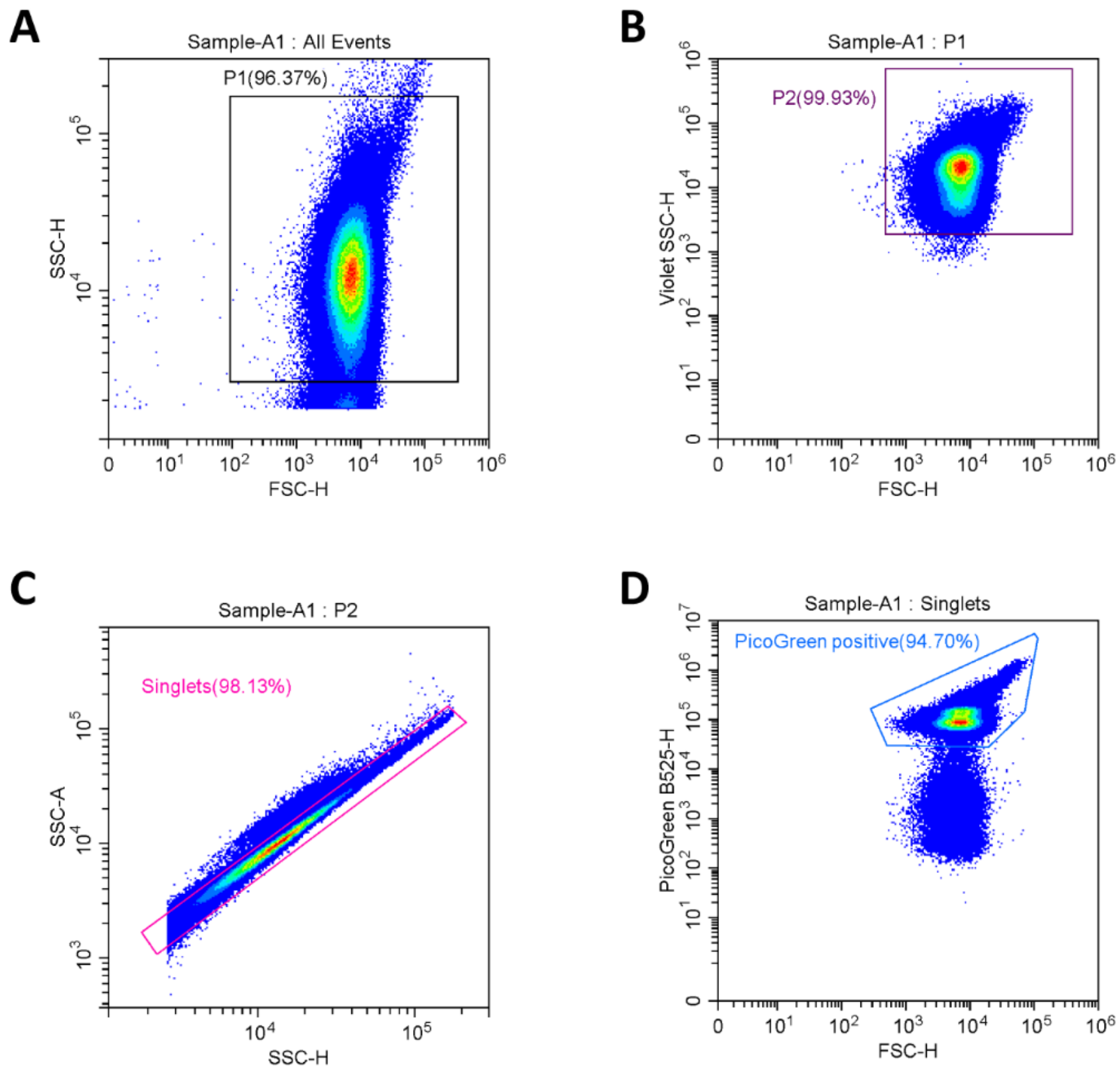

Figure S9. Gating strategy employed in the measurement of DNA content. A) The population of cells are gated from noise by plotting SSC-H against FSC-H using a blue laser (population P1). **B)** Cells are then more finely gated from noise by plotting the SSC-H obtained using a violet laser versus the same FSC-H (population P2). **C)** For population P2, SSC-H is then plotted against SSC-A and the Singlets population is gated from doublet events by gating on the diagonal. **D)** Finally, the intensity of the PicoGreen dye in the Singlets population is obtained and the positive cells for PicoGreen are gated from the rest. Histograms of DNA contents (see Figure S2 for an example) are then obtained from the PicoGreen positive population.

Table S1. List of bacterial strains used in this study, with related genotypes and sources.

**Strain**	**Characteristics**	**Source**
E. coli MG1655	K-12; F$^-$ l$^-$ *rph-1*	ATCC
E. coli DH5α	MG1655; *endA1 glnV44 thi-1 recA1 relA1 gyrA96 deoR nupG* f80d*lacZ*ΔM15 Δ(*lacZYA-argF)U169 hsdR17 (*r_K^- m_K^+)	New England Biolabs
E. coli DH5α x pCoRe-T	DH5α harbouring the pCoRe-T plasmid	This study
E. coli DH5α x pCoRe-Dcos	DH5α harbouring the pCoRe-Dcos plasmid	This study
E. coli pre-CoRTeDcos	MG1655; D*dnaAp*-*dnaA*::lox66/71-*pLtetO-1-tetR*-*dnaAcos*-lox66/71	This study
E. coli CoRTeDcos	Pre-CoRTeDcos; *dnaB*(G726>T)	This study

Table S2. Complete chemical composition of cultivation media used this study.

Identifier	Buffer/base composition	Carbon source	Supplement
Medium M1	K_2HPO_4 36.7 mM, NaCl 8.6 mM, Na_2HPO_4 47.8 mM, NH_4Cl 18.7 mM, $CaCl_2$ 0.5 mM, $MgCl_2$ 1 mM, $FeCl_3$ 13 µM, $ZnCl_2$ 6.2 µM, $CuCl_2$ 0.76 µM, $CoCl_2$ 0.42 µM, H_3BO_3 1.62 µM, $MnCl_2$ 0.081 µM	Glycerol 22 mM	/
Medium M2		Glucose 22 mM	Leu 0.6 mM, Ile 0.6 mM, Val 0.6 mM, thiamine 0.03 mM
Medium M3		Glucose 22 mM	Arg 1.15 mM, Asn 0.38 mM, Asp 0.15 mM, Cys 0.21 mM, Glu 0.14 mM, Gly 0.13 mM, His 0.1 mM, Ile 0.38 mM, Leu 0.38 mM, Lys 0.22 mM, Met 0.1 mM, Phe 0.09 mM, Pro 0.17 mM, Ser 0.29 mM, Thr 0.17 mM, Trp 0.025 mM, Tyr 0.11 mM, Val 0.17 mM
Medium M4	Pancreatic digest of casein 17 g/L, papaic digest of soybean 3 g/L, NaCl 85 mM, K_2HPO_4 14 mM	Glucose 22 mM	/

Table S3. List of plasmids used in this study.

**Plasmid**	**Characteristics**	**Source**
pSC020	*repA101*(Ts), *oriV, ampR, araC, P_{BAD}-lRed, lacIq, P_{Lac}-Cre*	(79)
pCoRe-T	pUC19 backbone; *ampR, lox66-cmR-lox71, pLtetO-1-tetR, BsaI*	This study
pCoRe-Dcos	pUC19 backbone; *ampR, BsaI, dnaAcos, lox66-kanR-lox71*	This study

Table S4. List of primers and chemically synthesised DNA used in this study. Sequences introducing homology arms for recombination into the genome of *E. coli* MG1655 are highlighted in bold.

Identifier	Sequence (5′-3′)	Used for
BG22166	cgagtggagtccgccatgtcactttcgctttggcagcagtgtcttgcccgattgcagg<br>atgagttaccagccacagaattcagtatgtggatacgcccattgcaggcggaactg<br>agcgataacacgctggccctgtacgcgccaaaccgttttgtcctcgattgggtacgg<br>gacaagtaccttaataatatcaatggactgctaaccagtttctgcggagcggatgcc<br>ccacagctgcgttttgaagtcggcaccaaaccggtgacgcaaacgccacaagcgg<br>cagtgacgagcaacgtcgcggcccctgcacaggtggcgcaaacgcagccgcaac<br>gtgctgcgccttctacgcgctcaggttgggataacgtcccggccccggcagaaccg<br>acctatcgttctaacgtaaacgtcaaacacacgtttgataacttcgttgaaggtaaat<br>ctaaccaactggcgcgcgcggcggctcgccTTgtggcggataaccctggcggtgc<br>ctataacccgttgttcctttatggcggcacgggtctgggtaaaactcacctgctgcat<br>gTTgtgggtaacggcattatggcgcgcaagccgaatgccaaagtggtttatatgc<br>actccgagcgctttgttcaggacatggttaaagccctgcaaaacaacgcgatcgaa<br>gagtttaaacgctactaccgttccgtagatgcactgctgatcgacgatattcagttttt<br>tgctaataaagaacgatctcaggaagagtttttcTATaccttcaacgccctgctgga<br>aggtaatcaacagatcattctcacctcggatcgcCACccgaaagagatcaacggc<br>gttgaggatcgtttgaaatcccgcttcggttggggactgactgtggcgatcgaaccg<br>ccagagctggaaacccgtgtggcgatcctgatgaaaaaggccgacgaaaacga<br>cattcgtttgccgggcgaagtggcgttctttatcgccaagcgtctacgatctaacgta<br>cgtgagctggaaggggcgctgaaccgcgtcattgccaatgccaactttaccggac<br>gggcgatcaccatcgacttcgtgcgtgaggcgctgcgcgacttgctggcattgcag<br>gaaaaactggtcaccatcgacaatattcagaagacggtggcggagtactacaag<br>atcaaagtcgcggatctcctttccaagcgtcgatcccgctcggtggcgcgtccgcgc<br>cagatggcgatggcgctggcgaaagagctgactaaccacagtctgccggagatt<br>ggcgatgcgtttggtggccgtgaccacacgacggtgcttcatgcctgccgtaagatc<br>gagcagttgcgtgaagagagccacgatatcaaagaagatttttcaaatttaatcag<br>aacattgtcatcgtaaacctat	Synthetic DNA of *dnaAcos*
BG22660	**gcctcgcggcaggatcgtttacacttagcgagttctggaaagtcctgtgg**gctagtt<br>attgctcagcggtgg	Amplified pCoRe-T fragment
BG20764	gcgtttcggtgatgacggtgaaaac	Amplified pCoRe-T fragment
BG21406	cgattcattaatgcagctggcacg	Amplified pCoRe-Dcos fragment
BG22661	**taaatgctcacgttctacggtaaatttcataggtttacgatgacaatgtt**ccgactgg<br>aaagctaccg	Amplified pCoRe-Dcos fragment
BG22467	cggcaatttccgcgccttc	Amplified *dnaA* locus in

		E. coli genome
BG22496	gacctcacaccagtggaaaccag	Amplified *dnaA* locus in *E. coli* genome
SJO416	ttcaccagatgggcaatcacttcgg	Amplified *oriC* in digital PCR
SJO417	acattgtggtggtgcataacggcatc	Amplified *oriC* in digital PCR
SJO432	atggatcttcctgctataccgccac	Amplified *ter* in digital PCR
SJO433	ggccaacatttgcgtccacagataag	Amplified *ter* in digital PCR

Table S5. Whole genome sequencing results of the strain used in this study. NC_000913.3 is the accession number of *E. coli* K-12 MG1655 reference genome.

**Strain**	**Reference genome**	**Mutations compared to reference**
E. coli MG1655	NC_000913.3	276’697 C→A (*mmuP* A458E); 1’873’031 IS1(–) + 9 bp (*dgcJ*); 4’296’381 IS +GC (intergenic)
E. coli pre-CoRTeDcos	MG1655	D*dnaAp-dnaA*::lox66/71-*pLtetO-1*-*tetR*-*dnaAcos*-lox66/71
E. coli CoRTeDcos	Pre-CoRTeDcos	4’265’708 G→T (*dnaB* M424I)

Table S6. Number of cells imaged per strain, condition and replicate to obtain cell size measurements. In the "Total number of cells" column, the three numbers between brackets represent the number of cells imaged in replicate 1, replicate 2 and replicate 3, respectively.

Strain	Medium	aTc (ng/mL)	Number of cells (replicates)
E. coli MG1655	M1	0	6227 (2213, 2387, 1627)
		10	6581 (2381, 2031, 2169)
	M2	0	7408 (2498, 2370, 2540)
		10	7448 (2947, 2245, 2256)
	M3	0	6674 (2592, 2067, 2015)
		10	6779 (2286, 2399, 2094)
	M4	0	6965 (2739, 2284, 1942)
		10	6991 (2609, 2406, 1976)
E. coli CoRTeDcos	M1	0	6313 (2097, 2121, 2095)
		0.5	7017 (2621, 2398, 1998)
		1	7108 (2588, 2453, 2067)
		2.5	6403 (2120, 2250, 2033)
		5	6582 (2150, 2036, 2396)
		10	6990 (2767, 1997, 2226)
		15	7057 (2118, 2094, 2845)
		20	6888 (2353, 2144, 2391)
	M2	0	4550 (1390, 1857, 1303)
		10	6740 (2063, 2325, 2352)
	M3	0	6611 (2852, 2205, 1554)
		0.5	6053 (2022, 2135, 1896)
		1	6385 (2316, 2096, 1973)
		2.5	7983 (2714, 2706, 2563)
		5	6496 (2207, 2427, 1862)
		10	6007 (1794, 2210, 2003)
		15	6530 (2659, 2305, 1566)
		20	6328 (2455, 1742, 2131)
	M4	0	7035 (2389, 2575, 2071)
		10	6906 (2493, 2173, 2240)

Table S7. Parameters used in the cell cycle simulations. With the exception of doubling time, all other parameters were left as in the original work (48) to recreate the established relationships.

Parameter	Values
Doubling time (τ)	20 min, 30 min, 60 min, 120 min
DnaA synthesis rate (α_A)	From 60 h^{-1} μm^{-3} to 3840 h^{-1} μm^{-3}
Used in titration and titration-extrusion models	
Critical DnaA free concentration ($[A_f^c]$)	10 μm^{-3}
Extruder synthesis rate (α_H)	180 h^{-1} μm^{-3}
Used in titration-switch and titration-switch-extrusion models	
Total DnaA concentration ($[D]_T$)	400 μm^{-3}
Lipids activation rate (α_l)	750 h^{-1}
DARS1 activation rate (α_{d1})	100 h^{-1}
DARS2 activation rate (α_{d2})	643 h^{-1}
datA deactivation rate (β_{datA})	600 h^{-1}
RIDA deactivation rate (β_{RIDA})	500 h^{-1}
Dissociation constant of DnaA activation/deactivation (K_D)	50 μm^{-3}
Dissociation constant of DnaA promoter (K_D^P)	400 μm^{-3}
Cooperativity of *dnaA* expression (n)	5
Extruder synthesis rate (α_H)	550 h-1 μm^{-3}
Critical DnaA-ATP concentration ($[A_{ATP}^{fc}]$)	200 μm^{-3}

Datasets

Data S1: Source data used to generate Figures 1-5 in the main text and Figures S1, S3-S6 and S8 in Supplementary Material.
